# Dataset of the PV surface temperature distribution when generating electricity (PV-On) and without generating electricity (PV-Off) using Halogen Solar Simulator

**DOI:** 10.1016/j.dib.2019.104578

**Published:** 2019-09-27

**Authors:** Erkata Yandri

**Affiliations:** Graduate School of Renewable Energy, Darma Persada University, Jl. Radin Inten 2, Pondok Kelapa, East Jakarta 13450, Indonesia

**Keywords:** Indoor experiment, Halogen solar simulator, PV surface temperature, PV operating temperature, PV electricity generation, PV open-closed circuit, Joule heating

## Abstract

These datasets present the Joule heating effect from our experimental series for the paper entitled, “Development and Experiment on the Performance of Collector with Polymeric Thermal Photovoltaic Thermal (PVT) Collector with Halogen Solar Simulator” [1]. The experiment was carried out before integrating the photovoltaic (PV) module part with the thermal (T) collector part into a hybrid PVT collector. The Joule heating effect was investigated from the surface temperature distribution of a PV module when generating electricity (PV-On). The irradiance was set to 1000 W/m^2^ using a pyranometer. After replacing by the PV module, the PV output has been set at the maximum power point (MPP) by setting the sliding variable resistor. The measurement took place at the rear side of the PV module to avoid the cable obstructing the light beam from the solar simulator, The thermocouples were installed at 10 points and connected to the data-logger. After steady-state in 30 minutes from starting the experiment, the measurements of the PV-On and PV-Off took place alternately 10 minutes by opening and closing the circuit. After completing the experiment, the light source was turned off for naturally cooling. These data is useful to provide an overview of PV surface temperature during PV-On and PV-Off. These datasets provide a more detailed picture of the temperature distribution on the PV surface to be more accurate in analyzing the performance of the PV module for simulation or field application. These datasets can be used as a reference for further improvements, especially in designing a more uniform solar simulator and understanding the operational modes of the PV module.

**Table:** Specifications Table

Subject	Energy
Specific subject area	Solar Energy
Type of data	Table
How data were acquired	Raw data were acquired by the data logger (Graphtec midi Logger GL220) during the indoor experiment and then transferred to Excel file for further analysis.
Data format	Raw and Analyzed
Parameters for data collection	These datasets were derived from indoor experiments using halogen solar simulators, with several controlled parameters, such as; data collection every 30 seconds/sampling, room temperature 26 °C, irradiance 1000 W/m^2^, closed room with normal air circulation and without lighting.
Description of data collection	These data were collected by a data-logger taken by thermocouples installed at 10 measurement points of the rear surface of the PV module. The data-logger was set to collect the data every 30 seconds for 60 minutes. The steady-state was achieved after 30 minutes from start, then the measurements of the PV-Off and PV-On was started alternately 10 minutes by opening and closing the circuit. After completing the experiment, the data was read and stored in Excel for further analysis.
Data source location	Kanagawa Institute of Technology, Atsugi, Kanagawa, Japan
Data accessibility	Data is provided within this article.
Related research article	Yandri E. Development and experiment on the performance of polymeric hybrid Photovoltaic Thermal (PVT) collector with halogen solar simulator. Sol Energy Mater Sol Cells 2019; 201:110066. https://doi.org/10.1016/j.solmat.2019.110066 [1].

**Table:** 

**Value of the data** • These datasets are useful to provide an overview of the behaviour and distribution of PV surface temperature when generating or not generating electricity. • With more measurement points, these datasets provide a more detailed picture of the temperature distribution on the PV surface of the module, such as from the middle to the edge, on the segment and the connecting line between segments, etc. • Using these datasets, researchers will be more accurate in analyzing the performance of the PV module with simulation or field application. • These datasets can be used as a reference for further improvements, especially in designing a more uniform solar simulator and understanding the operational modes of the PV module. • These datasets are very interesting to learn more about the effects of Joule heating in the integration of PV module with solar collectors into a hybrid photovoltaic thermal (PVT) collector.

## 1 Data

**Fig. 1:**
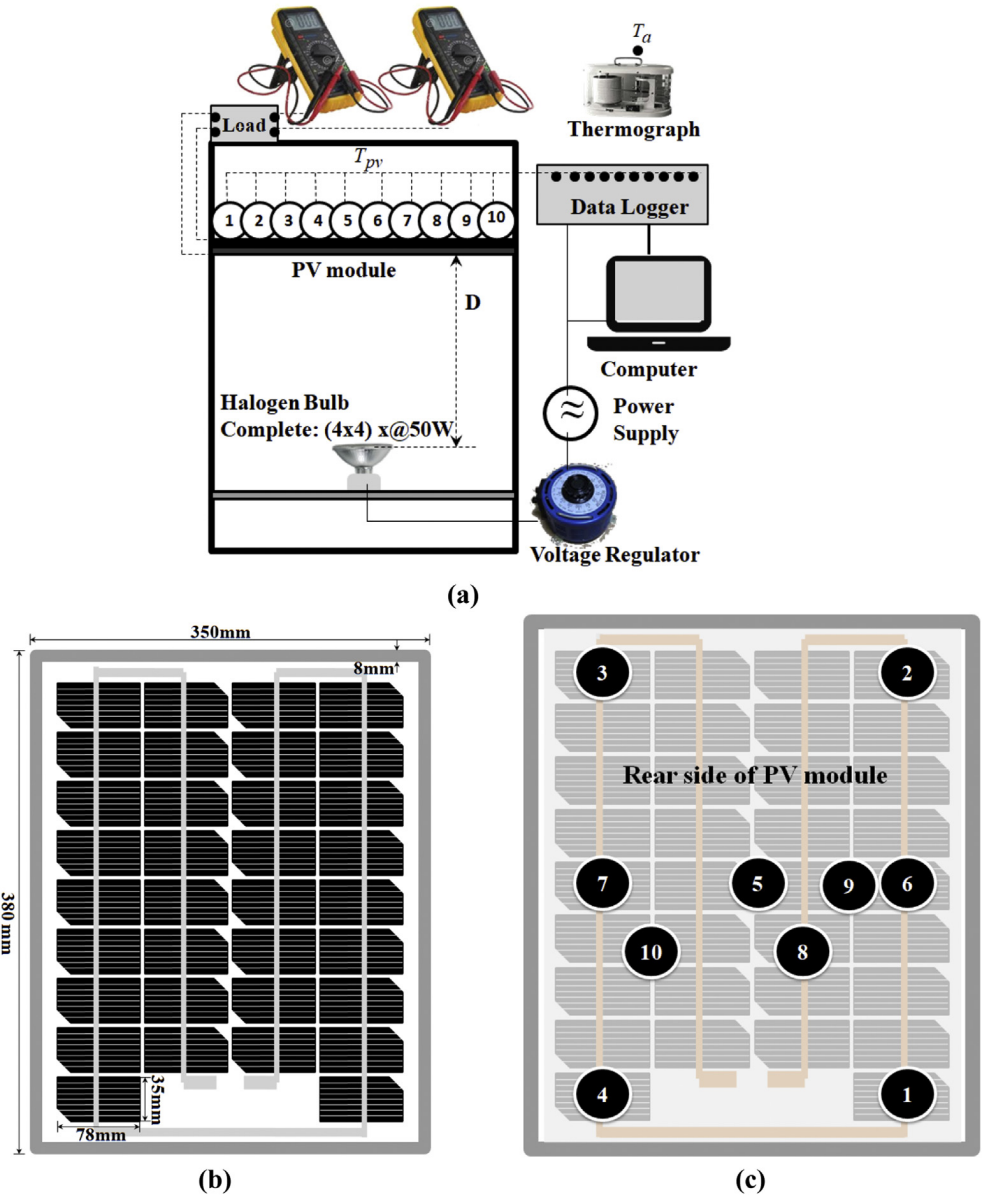
Experimental design, a). Complete setup of the experiment, b). The front side of the PV module, c). Measurement positions at the rear side of the PV module.

These datasets display the measurement results of photovoltaic (PV) surface temperature from an indoor experimental design, as shown in Fig. 1. Fig. 1(a) describes the complete setup of the experiment. Fig. 1(b) describes the front side of the PV module. Fig. 1(c) describes the detailed measurement positions at the rear side of the PV module. These datasets were taken from 10 measurement points, which represents the surface temperature of middle, edge and corner of the PV module. As a result, those PV surface temperatures can be seen in Table 1. The horizontal direction shows the PV surface temperature of 10 measurement points and the average, while the vertical direction is a measurement condition from PV-Off to PV-On alternately every 10 minutes in 1 hour. These datasets are useful in analyzing the PV surface temperature distribution of a PV module, including analyzing the Joule heating effect when generating electricity [2].

**Table 1:** PV surface temperature distribution during PV-On and PV-Off.

Minute	Mode	PV surface temperature, $T_{pv}$ (^o^C)										
		1	2	3	4	5	6	7	8	9	10	Average
00–10	PV-Off	36.11	35.90	37.07	36.22	39.90	36.41	36.35	38.76	37.24	38.52	37.25
11–20	PV-On	36.26	36.01	37.35	36.19	40.23	36.77	36.62	39.07	37.55	38.64	37.47
21–30	PV-Off	36.81	36.52	37.63	36.23	40.41	37.23	36.75	39.32	37.85	39.00	37.77
31–40	PV-On	37.02	36.70	37.77	36.46	40.65	37.53	36.91	39.54	38.13	39.19	37.99
41–50	PV-Off	36.86	36.87	37.99	36.66	40.80	37.65	37.01	39.73	38.32	39.37	38.14
51–60	PV-On	36.41	36.85	38.12	36.92	40.81	37.31	37.17	39.66	38.24	39.49	38.14

## 2 Experimental design, materials, and methods

Fig. 1 shows the detailed experimental design. As shown in Fig. 1(a), the complete setup of the experiment using a solar simulator, which consists of two main parts, ie: the solar module and the halogen light source. Regarding the solar simulator used; including the reasons, dimensions, construction, and characteristics, can refer to Ref. [3]. There is some important and related information. As explained, the dimensions of the simulator are 430 mm × 390 mm x 1000 mm, made of an aluminium frame. The optimum distance between the PV surface and the surface of light sources was set to D=32 cm. The light source consists of 16 halogen lamps (Ushio JDR50, 50 W/110 V) with a 4 × 4 array, mounted on an aluminium plate 380 mm × 350 mm, with a thickness of δ3 mm. The position and configuration of halogen lamps are fixed and can be switched on-off independently using its manual switch.

The data-logger (Graphtec midi Logger GL220, 10 channels) was programmed to collect the data every 30 seconds. Using a sliding manual voltage regulator (Matsunaga Mfg, Co. Ltd, Type SD-1310) to adjust the voltage input $V_{i}$ to the halogen simulator, the irradiance $I_{o}$ was set to 1000 W/m^2^. Due to the limited port on the data-logger, the room temperature $T_{r}$, considered as the ambient air temperature $T_{a}$ , was recorded by the room thermograph. The sensor of $T_{r}$ or $T_{a}$ was placed in the middle of the ceiling and set to 26 °C. As shown in Fig. 1(b) for the solar module part, we used the mono-crystalline PV module (GT434 type, KIS Solar Japan), with the outer dimensions of 380 × 350 mm. By calculating the area of 17 existing solar cell segments, the effective area of PV $A_{pv}$ is 0.091 m^2^.

As shown in Fig. 1(c), the surface temperature of the PV module $T_{pv}$ was measured at the rear side of the PV module. The reason is to avoid the measurement cable obstructing the light beam from the solar simulator. The measurement points are spread evenly at 10 points to get a clearer picture of the data. Those points represent the middle, edge and corner of the PV module. The indoor experiment can give more controls to the external parameters, such as ambient air temperature $T_{a}$, irradiance $I_{o}$, wind speed $V_{w}$ [4]. This condition is expected in investigating the effect of Joule heating on the thermal performance of the PVT collector during electricity generation [2]. In theory, the output of a PV module depends on the operating temperature of the module [5].

To make the accuracy, the irradiance $I_{o}$ was measured using a pyranometer (MS-42, Eko) before starting the experiment. After that, the pyranometer was replaced by the PV module. To get the optimal Joule heating effect, the PV output of PV module has been set at the maximum power point (MPP) by setting the sliding variable resistor. We started the experiment at $T_{pv}\approx T_{a}$. After 30 minutes of starting the experiment, the steady-state has been reached. Then, the measurements for PV-Off and PV-On took place alternately with 10 minutes each by opening and closing the circuit.

To protect the PV module not to be exposed to the halogen rays for too long, the collected data was carried out for 60 minutes. Then, the light source was turned off. The PV module is cooled naturally by air. Finally, the experiment was complete. The data-logger stored the CSV file format in tabular data, such as a spreadsheet or database. The data can be exported to Microsoft Excel for further analysis. For simplification, we present the data of PV surface temperature distribution in the average of 10 minutes per mode for each measurement point, as shown in Table 1.
